# Design, Synthesis, and Antitumor Activity Evaluation of Artemisinin Bivalent Ligands

**DOI:** 10.3390/molecules29020409

**Published:** 2024-01-15

**Authors:** Hui Zhong, Qi Jiang, Cong Wu, Huanghe Yu, Bin Li, Xudong Zhou, Ronggeng Fu, Wei Wang, Wenbing Sheng

**Affiliations:** 1 School of Pharmacy, Hunan University of Chinese Medicine, Changsha 410208, China; zhonghui@stu.hnucm.edu.cn (H.Z.); jiang7_1115@163.com (Q.J.); cw191436@163.com (C.W.); yhh@hnucm.edu.cn (H.Y.); libin@hnucm.edu.cn (B.L.); xudongzhou999@163.com (X.Z.); furonggeng@hnucm.edu.cn (R.F.); 2 TCM and Ethnomedicine Innovation and Development International Laboratory, Hunan University of Chinese Medicine, Changsha 410208, China

**Keywords:** artemisinin, bivalent ligands, multi-target molecular docking, antitumor target protein, antitumor activity

## Abstract

Five artemisinin bivalent ligands molecules **4a**–**4e** were designed, synthesized, and confirmed by ^1^H NMR, ^13^C NMR, and low-resolution mass spectrometry, and the bioactivities of the target compounds were investigated against four human tumor cell lines in vitro, including BGC-823, HepG-2, MCF-7, and HCT-116. The results showed **4a**, **4d,** and **4e** exhibited significantly tumor cell inhibitory activity compared with the artemisinin and dihydroartemisinin; compound **4e** has good biological activity inhibiting BGC-823 with an IC_50_ value of 8.30 μmol/L. Then, the good correlations with biological results were validated by molecular docking through the established bivalent ligands multi-target model, which showed that **4e** could bind well with the antitumor protein MMP-9.

## 1. Introduction

Artemisinin (ART) is a novel sesquiterpene containing an endoperoxide structure, with significant antimalarial activity of a fast-acting, high-efficiency, and low-toxicity nature [1]. Some artemisinin-based derivatives have also become excellent clinical drugs such as dihydroartemisinin (DHM), artemether, artesunate, and so on [2,3,4]. During the research of structural modification, artemisinin derivatives have shown superior antitumor activity [5], such as against ovarian cancer [6,7], leukemia [8], liver cancer, colon cancer [9], bladder cancer, and breast cancer [10]. Dihydroartemisinin and dihydroartemisitene acetal dimers were synthesized and tested for their antitumor activity against human tumor 60 cell lines, and the GI_50_ concentration ranged from 0.019 to 8.7 μmol/L against non-small-cell lung cancer, central nervous system tumors, leukemia cells, and other cancer cells [11]. Chadwick et al. [12] synthesized a series of C-10 carba artemisinin dimers with good inhibitory effects on human promyelocytic leukaemia HL-60 cells with an IC_50_ value in the micromolar to nanomolar range.

Cancer is a serious threat to human health [13]. Despite remarkable progress in cancer prevention and treatment over the past decades, traditional tumor chemotherapeutic drugs often fail to distinguish between tumor cells and normal cells, and the development of new safe and effective drugs for cancer treatment remains extremely challenging [14]. Natural products provide a rich source of leads for the discovery of new anticancer drugs [15].

“Bivalent ligands” molecules are antitumor star molecules, consisting of a variable-sized linker group connecting two pharmacodynamic groups [16]. Bivalent ligands show the advantages of enhanced binding affinity and significantly improved targeting to a tumor cell [17,18,19], which provides new ideas for drug design. Previous studies have shown that artemisinin bivalent ligands have higher biological activity than the corresponding monomers [20,21,22,23,24]. And appropriate substitution in the linker of two artemisinin units can improve the antitumor activity of the analogs [4]. Porphyrins and porphyrin derivatives are important functional molecules for cancer therapy [25,26,27]. Huang et al. bridged the natural product with porphyrin and the synthesized derivatives showed a promising cleavage effect on pBR322 plasmid DNA [28,29]. Heterocycles have always been key elements in medicinal chemistry as well as being found in the structure of numerous drugs, drug candidates, and biologically active molecules [30].

**Figure 1:**
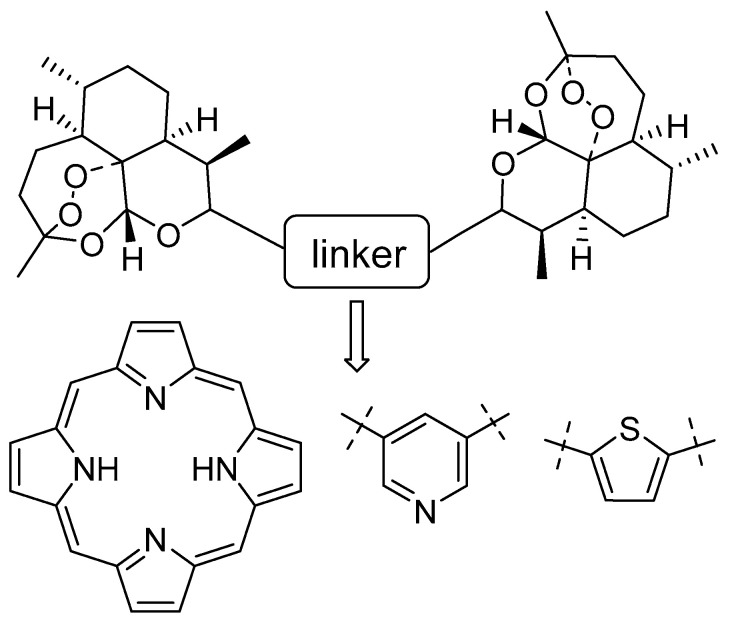
Design of compounds.

Guided and impelled by these results, five artemisinin bivalent ligands linked by different molecules were designed and synthesized with the purpose of enhancing the targeting and antitumor activity of artemisinin (Figure 1); their anti-proliferative activities were evaluated and the potential mechanisms were investigated preliminarily on representative tumor cells by Western blot assay. Furthermore, molecular docking models were performed to validate the artemisinin-multitarget molecules via LeDock.win32 software.

## 2. Results and Discussion

### 2.1. Chemistry

**Scheme 1:**
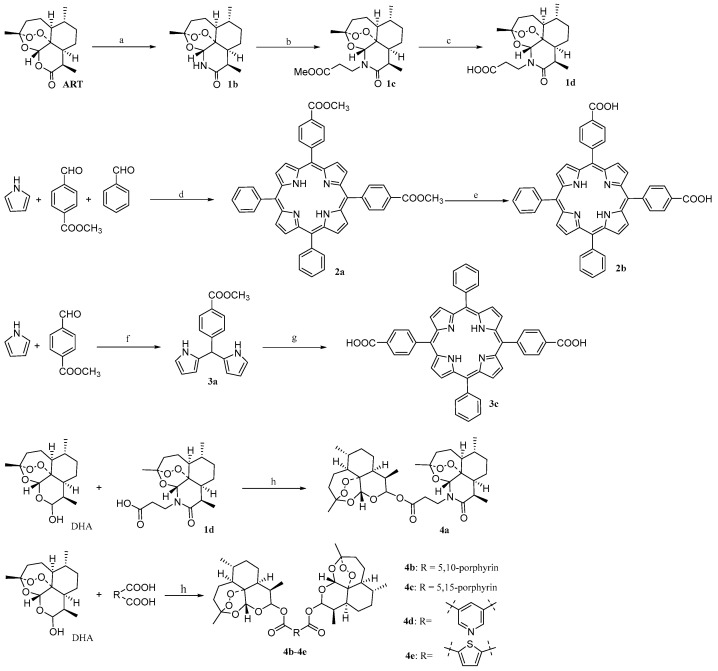
Synthetic route of bivalent ligands. Reagents and conditions: (a) i: NH_3_(aq.), THF/MeOH, −10 °C,10 h; ii: *p*-TsOH, DCM, r.t., 12 h, 79%; (b) methyl acrylate, NaOH, THF, r.t., 80%; (c) NaOH, THF/MeOH, 66 °C, 12 h, 93%; (d) propionic acid, 140 °C, 33%; (e) NaOH, THF/MeOH, 66 °C, 12 h, 92%; (f) N_2_, CF_3_COOH, r.t., 41%; (g) i: benzaldehyde, N_2_, DCM, r.t., 2 h, ii: Chloranil, r.t., 12 h, 16%, iii: NaOH, THF, 66 °C,12 h, 91%; (h) DCC or EDCI·HCl, DMAP, N_2_, DCM or DMF, r.t., 24 h.

Artemisinin bivalent ligands **4a**–**4e** were catalyzed by 4-dimethylaminopyridine (DMAP) and imine according to the synthetic routes outlined in Scheme 1. The yields of compounds **4a**–**4c** were improved as the amounts of DMAP used increased. Compound **4d** was generated by the reaction in the presence of DMAP and 1,3-dicyclohexylcarbodiimide (DCC), and it was difficult to purify for the by-product 1, 3-dicyclohexylurea (DCU) as well as being produced in low yield, as was **4e**. Yet, these disadvantages can be effectively overcome by changing the condensing agents to 1-ethyl-3-(3-dimethylaminopropyl) carbodiimide hydrochloride (EDCI·HCl) instead of DCC.

### 2.2. Biological Evaluation

#### 2.2.1. Screening of Anti-Cancer Activity

Anti-proliferation activity of **4a**–**4e** in vitro was evaluated by four tumor cell lines, including MCF-7, HepG-2, HCT-116, and BGC-823, using the MTT assay, and the results were shown in Table 1.

Compared to the parent compound ART, compounds **4a**, **4d**, and **4e** displayed promising antiproliferative activity against the abovementioned tumor cells lines. Among them, compound **4a** showed better inhibitory activity against each tumor cell line, exhibiting 2–4-fold increased activity compared to ART, indicating it possesses a broad spectrum of antitumor activity. Compounds **4d** and **4e**, which possessed a heterocyclic ring as the linker, improved the inhibitory activity against some tumor cell lines. In particular, compound **4e** showed the best inhibitory activity against BGC-823 with an IC_50_ value of 8.30 μmol/L. Unfortunately, the activity of compounds **4b** and **4c** were not as good as expected, proving that the introduction of bulky substituents on the linker had a detrimental effect on the activity. It may be related to the permeability of the molecule, which faces difficulty in penetrating through the cell membrane due to the large structural skeleton.

Preliminary structure–activity relationship (SAR) studies demonstrated that the linker between two artemisinin molecules had an effect on their antitumor activity. The use of linkers with small backbones or high polarity is more helpful in improving antitumor activity. The SAR studies suggest that other heterocycles, such as thiazole and pyrimidine, can be used as linkers to further investigate the effect of the type of linker on antitumor activity in future studies.

#### 2.2.2. Western Blot Analysis

**Figure 2:**
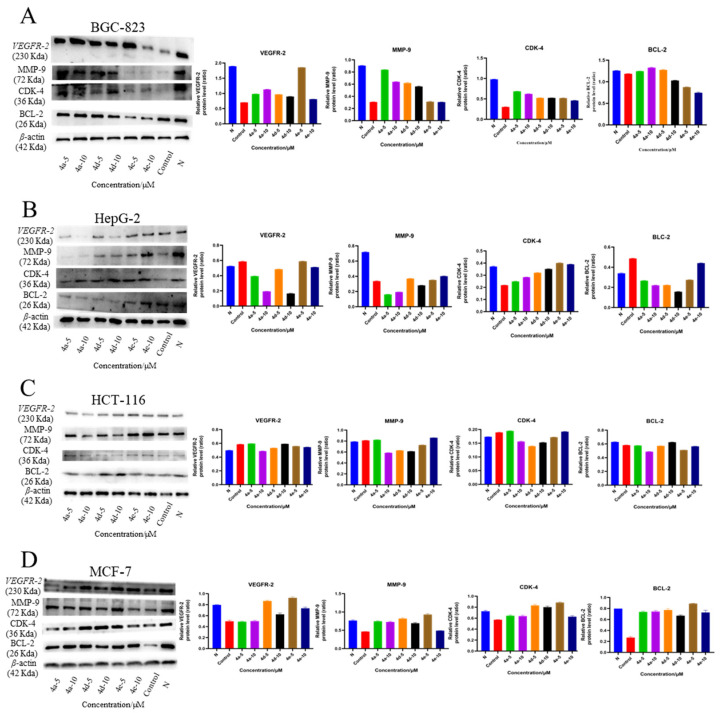
Effects of compound **4a**, **4d**, and **4e** on the proteins of VEGFR-2, MMP-9, CDK-4, and BCL-2 in the four tumor cells lines. (**A**) BGC-823 cells. (**B**) HepG-2 cells. (**C**) HCT-116 cells. (**D**) MCF-7 cells. The cells were treated with compounds for 48 h, and the expression level of related proteins was detected by Western blot.

The latest research has revealed that artemisinin can work on multiple targets and affect multiple signaling pathways against cancer cells, such as inducing apoptosis, triggering ferroptotic cell death, inducing autophagy, and causing cell cycle arrest [31,32]. In this study, we further evaluated the effects of artemisinin derivatives on apoptosis-related proteins in the four tumor cell lines. As shown by Western blotting in Figure 2, the effect of derivatives on the expression of different apoptotic proteins is variable in diverse tumor cells.

The Western blot assay results showed that compounds **4a**, **4d**, and **4e** effectively decreased the protein levels of apoptosis-related proteins BCL-2, CDK-4, MMP-9, and VEGFR-2 in BGC-823 and HepG-2 cells (Figure 2A,B). In particular, compound **4e** reduced the expression of the four apoptosis-associated proteins in BGC-823 cells more completely. Compounds **4a** and **4d** significantly inhibited the expression of protein VEGFR-2 in HepG-2 in a dose-dependent manner. However, all three of the tested compounds exhibited no major differences in the levels of expression of the four proteins over a 48-hour period in HCT-116 and MCF-7 cells (Figure 2C,D). Positive control Paclitaxel downregulated the protein level of CDK-4, MMP-9, and VEGFR-2 in the BGC-823 cells, decreased the expression of CDK-4 and MMP-9 in HepG-2, and reduced the expression of BCL-2, CDK-4, MMP-9, and VEGFR-2 in MCF-7 (Figure 2A–D).

#### 2.2.3. Molecular Docking

Docking studies were performed to investigate the molecular binding patterns of artemisinin divalent ligands **4a**–**4e** in the active pockets of the crystal structures of anticancer targets. The targets of the divalent ligand **4a**–**4e** used for docking analysis are BCL-2, CDK-4, VEGFR-2, and MMP-9, which are widely noted to contribute to cellular proliferation and apoptosis regulation, cell cycle progression, transcriptional regulation, DNA damage repair, stem cell self-renewal, and antiangiogenic effects [33,34,35]. The binding of ligands at the active site of the target protein suggests the possibility that ligands may have the ability to direct functional changes in target molecules. Ligand–target protein interaction is also decoded in terms of interacting amino acid residues, hydrogen bonding, docking energy analysis, and comparison of active site amino acid residues and possible binding sites.

**Figure 3:**
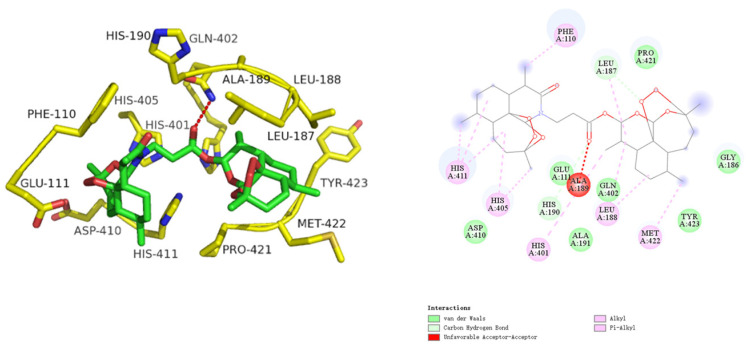
The 3D/2D binding modes/interactions of bivalent ligand **4a** into the active site MMP-9.

MMP-9 (PDB ID: 2OVZ) is identified as the target protein for anticancer reagents. Upon evaluation of the docking results, it is clear that bivalent ligand **4a** can fit perfectly into the binding pocket of MMP-9, demonstrating good uniformity between the in vitro MMP-9 screening and the in silico prediction. The docking results of bivalent ligand **4a** exhibited that it engaged in the formation of hydrogen bonds with GLU111 and LEU187 amino acid residues (Figure 3). Additionally, bivalent ligand **4a** is involved in numerous hydrophobic interactions, such as six Pi alkyl interactions with PHE110, HIS401, HIS405, HIS411, LEU188 and MET422. But, it forms an unfavorable acceptor–acceptor with ALA189.

**Figure 4:**
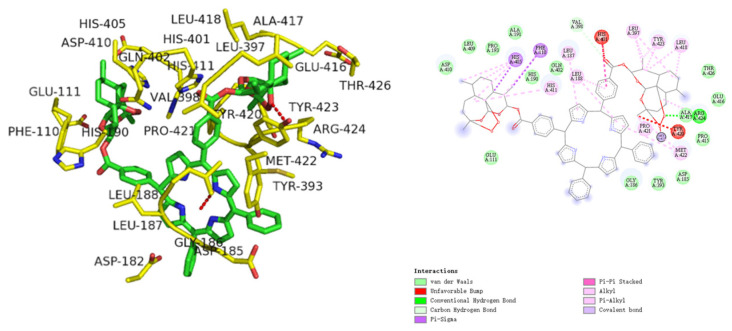
The 3D/2D binding modes/interactions of bivalent ligand **4b** into the active site MMP-9.

The results of the present in silico docking simulation revealed the significant bivalent ligand **4b**, which showed good hydrogen bonding interactions with the most important residues in the active site pockets of the proteins. The best confirmation of bivalent ligand **4b** with MMP-9 showed hydrogen bonds with ARG424 amino acid residues, as well as several hydrophobic interactions including Pi-sigma interaction with HIS405 and PHE110, Pi–Pi stacked interaction with MET422, Pi-sigma interaction with Leu607, carbon hydrogen bonding with VAL398, and Pi alkyl interaction (8) with HIS411, LEU187, LEU188, LEU397, LEU418, TYR423, PRO421, and MET422, as depicted in Figure 4.

**Figure 5:**
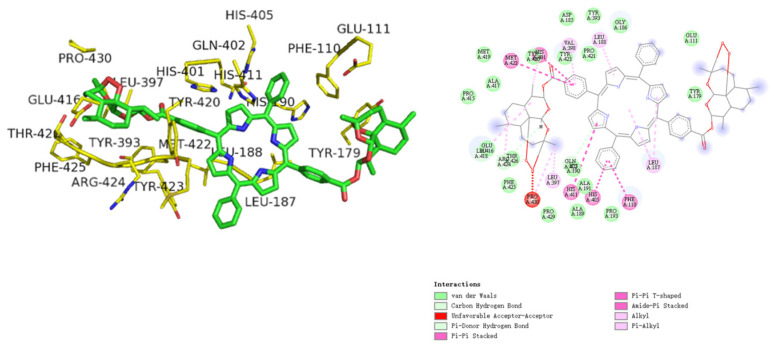
The 3D/2D binding modes/interactions of bivalent ligand **4c** into the active site MMP-9.

The results of the present in silico docking simulations show the strongest docking binding energy of the divalent ligand **4c** to MMP-9, which exhibits good multiple interactions with the most important residues in the active site pocket of the protein. The best confirmation of bivalent ligand **4c** with MMP-9 showed several hydrophobic interactions, including Pi–Pi T-shaped interaction with HIS401, Pi–Pi stacked interaction (5) with MET422, HIS401, HIS405, HIS411, and PHE110, carbon hydrogen bond (2) with ARG424 and GLN402, amide–Pi stacked interaction with (5) MET422, HIS401, HIS405, HIS411, and PHE110, and Pi alkyl interaction (8) with HIS401, LEU187, LEU188, LEU397, LEU418, ARG424, PRO430, and VAL398, as depicted in Figure 5.

**Figure 6:**
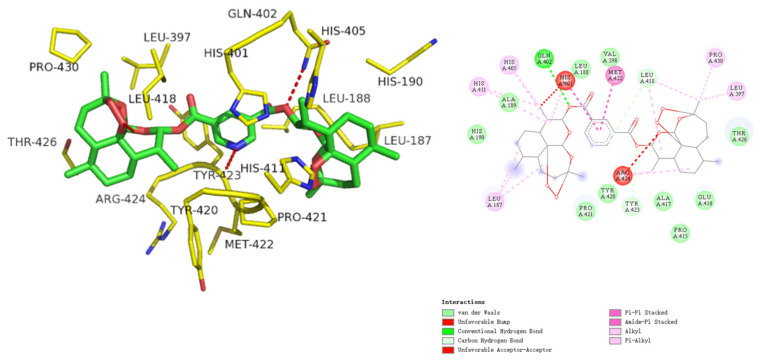
The 3D/2D binding modes/interactions of bivalent ligand **4d** into the active site MMP-9.

Docking results show strong docking binding of **4d** to MMP-9 and the presence of multiple interaction forces (Figure 6). The docking results of bivalent ligand **4d** exhibited that it engaged in the formation of H bonds with GLN402 amino acid residues. Also, it was engaged in the formation of many hydrophobic interactions such as two carbon hydrogen bonds with LEU418 and TYR423, Pi–Pi stacked interaction (2) with HIS401 and MET422, amide–Pi stacked interaction with (2) HIS401 and MET422, and Pi alkyl interaction (8) with HIS401, HIS405, HIS411, LEU187, LEU397, LEU418, ARG424, and PRO430.

**Figure 7:**
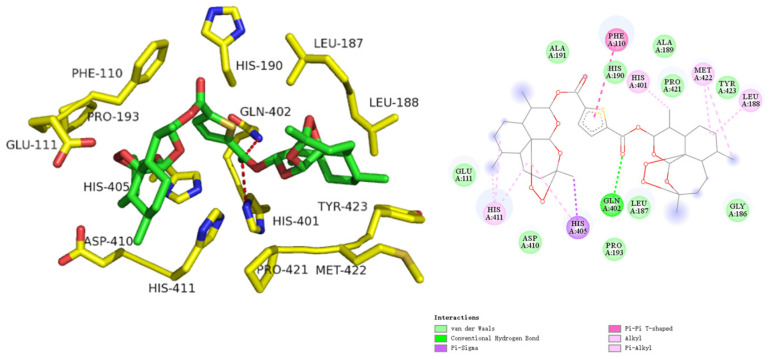
The 3D/2D binding modes/interactions of bivalent ligand **4e** into the active site MMP-9.

Analysis of the docking results revealed that the docking studies were consistent with the antitumor cell experiment. From the inspection of the docking results, bivalent ligand **4e** can fit perfectly into the catalytic binding pocket of MMP-9. As presented in Figure 7, bivalent ligand **4e** was incorporated in the formation of one hydrogen bond with GLN402 and Pi–Pi T-shaped interaction with PHE110, as well as Pi alkyl interaction (5) with HIS401, HIS405, HIS411, LEU188, and MET422.

Hydrogen bonding was also evaluated for the interaction of bivalent ligands **4a**–**4e** with these four targets. The amino acid residues involved in hydrogen bonding at the binding sites of the bivalent ligands **4a**–**4e** in BCL-2, CDK-4, VEGFR-2, and MMP-9 are summarized in Table 2. The total binding strength is the result of many types of bonding, including ionic, hydrophobic interactions and van der Waals forces, although hydrogen bonding is the major contributor. Hydrogen bonding also depends on the composition and three-dimensional arrangement of the contacting amino acid residues at the prominent and active binding sites.

Binding energies and theoretical inhibition constants (Ki) of artemisinin, dihydroartemisinin, and compounds **4a**–**4e** within the binding sites of BCL-2, CDK-4, VEGFR-2, and MMP-9 are shown in Table 3.

In Table 3, the binding energies of the target compounds with each target protein were significantly stronger than that of ART and DHA, except for compound **4c**, which showed lower binding activity to the VEGFR-2 protein than ART and DHA. The results were similar to those of the antitumor cell proliferation assay, indicating that compounds **4a**–**4e** have good interaction power with the four target proteins. However, the cellular activity of the target compounds **4b** and **4c** was opposite to the docking activity, probably because the structural backbone of the compounds was too large to penetrate the cell membrane and could not eventually intervene to inhibit the tumor cells.

## 3. Materials and Methods

### 3.1. Chemistry

The commonly used reagents were purchased from Sinopharm Group Chemical Reagent Co., Ltd. and used without further purification. Thin-layer chromatography (TLC, silica gel HSGF254, Yantai Jiang you Silicone Development Co., Ltd. (Yantai, Shandong, China)) was used to monitor for completeness of the reaction visualized by UV light (λ = 254 nm or λ = 365 nm). The target compounds were purified by silica gel column chromatography. NMR spectra were recorded on the Bruker Avance III spectrometer at 600 MHz for ^1^H NMR and 150 MHz for ^13^C NMR, with tetramethylsilane (TMS) as the internal standard, and DMSO-d6, CDCl_3_, or CD_3_OD as the solvent. Coupling constant (*J*) values were estimated in hertz (Hz). Splitting patterns are designated as follows: s, singlet; br s, broad singlet; d, doublet; t, triplet; q, quartet; dd, doublet of doublet; m, multiplet. Mass spectra were measured on an LCMS 6400 Series Triple Quadrupole Mass Spectrometer (Agilent). Intermediates **1a**–**1d**, **2a**, **2b**, **3a**, and **3c** were synthesized according to the references [36,37,38,39], and the detailed synthesis process and details can be seen in Supplementary Materials.

#### 3.1.1. Synthesis of Artemisinin Bivalent Ligand (**4a**)

In a 25 mL round-bottomed flask were placed **1d** (35 mg, 0.1 mmol), anhydrous DCM (5 mL), DCC (45 mg, 0.22 mmol), DMAP (27 mg, 0.22 mmol), and DHA (63 mg, 0.22 mmol). The mixture was protected by N_2_ at room temperature and monitored by TLC. After completion of the reaction, the reaction mixture was quenched by the addition of distilled water (10 mL), extracted with ethyl acetate, washed sequentially with saturated saline and distilled water, then dried with anhydrous Na_2_SO_4_ and distilled under reduced pressure to remove the solvent and purified by silica gel chromatography with ethyl acetate-petroleum ether (1:5) as eluent to afford **4a**.

**4a(C_33_H_49_NO_10_)**: white solid (45.2 mg, isolated yield 73%), ^1^H NMR (600 MHz, CDCl_3_) *δ* 5.78 (d, *J* = 9.9 Hz, 1H), 5.43 (s, 1H), 5.28 (s, 1H), 3.83–3.67 (m, 2H), 3.31–3.23 (m, 1H), 2.89–2.75 (m, 1H), 2.70 (ddd, *J* = 16.9, 9.3, 5.3 Hz, 1H), 2.59–2.48 (m, 1H), 2.46–2.32 (m, 2H), 2.06–1.95 (m, 4H), 1.88 (ddd, *J* = 10.0, 6.4, 3.2 Hz, 1H), 1.82–1.42 (m, 14H), 1.43 (s, 3H), 1.36 (s, 3H), 1.12 (d, *J* = 7.3 Hz, 3H), 0.99 (d, *J* = 6.4 Hz, 4H), 0.96 (d, *J* = 6.1 Hz, 3H), 0.84 (d, *J* = 7.1 Hz, 3H); ^13^C NMR (151 MHz, CDCl_3_) *δ* 171.89, 171.12, 104.93, 104.58, 91.97, 91.62, 80.25, 78.65, 51.67, 51.48, 45.79, 45.34, 37.91, 37.65, 37.39, 36.74, 36.33, 34.20, 33.75, 33.21, 32.31, 31.84, 26.10, 25.58, 25.18, 24.70, 22.88, 22.11, 20.36, 19.90, 19.30, 12.95, 12.26. LRMS *m*/*z*: [M]=619.34, [M+Na] ^+^ =642.20.

#### 3.1.2. Synthesis of Artemisinin Bivalent Ligand (**4b**)

In a 25 mL round-bottomed flask were placed **2b** (70 mg, 0.1 mmol), anhydrous DCM (5 mL), DCC (45 mg, 0.22 mmol), DMAP (27 mg, 0.22 mmol), and DHA (63 mg, 0.22 mmol). The mixture was protected by N_2_ at room temperature and monitored by TLC. After completion of the reaction, the reaction mixture was quenched by the addition of distilled water (10 mL), extracted with ethyl acetate, washed sequentially with saturated saline and distilled water, then dried with anhydrous Na_2_SO_4_ and distilled under reduced pressure to remove the solvent and purified by silica gel chromatography with dichloromethane as eluent to afford **4b**.

**4b(C_76_H_74_N_4_O_12_)**: purple solid (65.4 mg, isolated yield 53%), ^1^H NMR (600 MHz, CDCl_3_) *δ* 8.85 (d, *J* = 4.5 Hz, 2H), 8.78 (d, *J* = 4.7 Hz, 2H), 8.67 (d, *J* = 7.8 Hz, 2H), 8.58 (d, *J* = 6.1 Hz, 4H), 8.50 (d, *J* = 4.1 Hz, 2H), 8.27 (d, *J* = 7.8 Hz, 2H), 8.21 (dd, *J* = 7.2, 5.3 Hz, 4H), 7.97 (d, *J* = 7.8 Hz, 4H), 7.83–7.74 (m, 4H), 6.25 (d, *J* = 8.5 Hz, 2H), 5.70 (s, 2H), 2.20–2.11 (m, 2H), 2.09–2.00 (m, 2H), 1.96–1.88 (m, 6H), 1.70 (dt, *J* = 13.5, 3.9 Hz, 4H), 1.56 (s, 6H), 1.60–1.26 (m, 10H), 1.25 (d, *J* = 7.0 Hz, 6H), 0.88 (d, *J* = 7.1 Hz, 6H), −2.80 (s, 2H). LRMS *m/z*: [M] =1234.53.

#### 3.1.3. Synthesis of artemisinin bivalent ligand (**4c**)

Following the same procedure for **4a**, **3c** (70 mg, 0.1 mmol), DCC (45 mg, 0.22 mmol), DMAP (27 mg, 0.22 mmol), and DHA (63 mg, 0.22 mmol) were reacted to produce **4c**.

**4c(C_76_H_74_N_4_O_12_)**: purple solid (58.0 mg, isolated yield 47%), ^1^H NMR (600 MHz, CDCl_3_) *δ* 8.88 (d, *J* = 11.1 Hz, 4H), 8.80 (d, *J* = 6.9 Hz, 4H), 8.29 (d, *J* = 7.8 Hz, 4H), 8.23 (d, *J* = 6.7 Hz, 4H), 7.99 (d, *J* = 7.9 Hz, 4H), 7.84–7.75 (m, 6H), 6.28 (d, *J* = 7.1 Hz, 2H), 5.71 (s, 2H), 2.26–2.13 (m, 2H), 2.02 (dt, *J* = 13.1, 4.4 Hz, 2H), 1.96–1.90 (m, 6H), 1.84–1.76 (m, 4H), 1.55 (s, 6H), 1.66–1.27 (m, 10H), 1.22 (d, *J* = 7.2 Hz, 6H), 0.96 (d, *J* = 5.6 Hz, 6H), −2.78 (s, 2H). LRMS *m/z*: [M] =1234.53.

#### 3.1.4. Synthesis of Artemisinin Bivalent Ligand (**4d**)

To a mixture of pyridine-3,5-dicarboxylic acid (25 mg, 0.15 mmol) and anhydrous DMF (5 mL) was added EDCI·HCl (0.36 mmol), DMAP (0.36 mmol), and DHA (0.36 mmol), which was then protected by N_2_ at room temperature. The reaction was monitored by TLC. The slurry was quenched by the addition of distilled water (10 mL), extracted with ethyl acetate, washed sequentially with saturated saline and distilled water, dried with anhydrous Na_2_SO_4_, distilled in vacuo to remove the solvent, and purified by silica gel chromatography with ethyl acetate-dichloromethane (1:5) as eluent to obtain **4d**.

**4d(C_37_H_49_NO_12_)**: white solid (66.1 mg, isolated yield 63%), ^1^H NMR (600 MHz, CDCl_3_) *δ* 9.46 (d, *J* = 2.1 Hz, 2H), 8.98 (t, *J* = 2.1 Hz, 1H), 6.04 (d, *J* = 9.9 Hz, 2H), 5.54 (s, 2H), 2.85–2.76 (m, 2H), 2.39 (td, *J* = 14.0, 3.8 Hz, 2H), 2.08–2.02 (m, 2H), 1.92 (dq, *J* = 10.2, 3.3 Hz, 2H), 1.83 (dd, *J* = 13.6, 3.9 Hz, 2H), 1.76 (dd, *J* = 13.4, 3.3 Hz, 2H), 1.71 (dt, *J* = 13.8, 4.5 Hz, 2H), 1.43 (s, 6H), 1.51–1.24 (m, 8H), 1.09–1.01 (m, 2H), 0.99 (d, *J* = 6.1 Hz, 6H), 0.94 (d, *J* = 7.2 Hz, 6H); ^13^C NMR (151 MHz, CDCl_3_) *δ* 163.34, 154.91, 139.11, 125.83, 104.69, 93.47, 91.84, 80.23, 51.69, 45.39, 37.41, 36.33, 34.19, 31.94, 26.05, 24.70, 22.16, 20.37, 12.40. LRMS *m/z*: [M] = 699.33, [M+H]^−^ = 700.20.

#### 3.1.5. Synthesis of Artemisinin Bivalent Ligand (**4e**)

Following the same procedure for **4d**, thiophene-2,5-dicarboxylic acid (25 mg, 0.15 mmol), EDCI·HCl (69 mg, 0.36 mmol), DMAP (44 mg, 0.36 mmol), and DHA (102 mg, 0.36 mmol) were reacted to produce **4e**.

**4e(C_36_H_48_O_12_S)**: white solid (44.4 mg, isolated yield 42%), ^1^H NMR (600 MHz, CDCl_3_) *δ* 7.81 (s, 2H), 5.91 (d, *J* = 9.8 Hz, 2H), 5.51 (s, 2H), 2.72 (ddd, *J* = 9.7, 7.1, 4.4 Hz, 2H), 2.38 (td, *J* = 13.9, 3.9 Hz, 2H), 2.07–1.97 (m, 2H), 1.90 (dq, *J* = 9.9, 3.2 Hz, 2H), 1.81 (dd, *J* = 13.8, 3.9 Hz, 2H), 1.74 (dd, *J* = 13.4, 3.3 Hz, 2H), 1.68 (dt, *J* = 13.7, 4.5 Hz, 2H), 1.42 (s, 6H), 1.52–1.23 (m, 8H), 1.06–0.99 (m, 2H), 0.97 (d, *J* = 6.1 Hz, 6H), 0.92 (d, *J* = 7.1 Hz, 6H); ^13^C NMR (151 MHz, CDCl_3_) *δ* 160.43, 139.49, 133.44, 104.16, 92.81, 92.21, 80.21, 51.69, 45.34, 37.37, 36.32, 34.18, 31.95, 26.05, 24.67, 22.13, 20.35, 12.28. LRMS *m/z*: [M] = 704.29, [M − H]^−^ = 703.70.

### 3.2. Biological Evaluation

#### 3.2.1. Cell Culture Conditions

Both HepG-2 cells and BGC-823 cells were cultured in DMEM, while MCF-7 cells were cultured in MEM and HCT-116 cells were cultured in McCoy5A in a 37 °C, 5% CO_2_ humidified environment.

#### 3.2.2. Cytotoxicity In Vitro

Cell cytotoxicity was determined by methyl thiazolyl tetrazolium (MTT) assay. Four tumor cell lines, MCF-7, HepG-2, HCT-116, and BGC-823, were inoculated at 5 × 10^3^ cells per well in a 96-well plate. The cells were incubated with compounds for 48 h at five different concentrations. Then, the cells were incubated with 5 mg/mL MTT reagent and incubated for 4 h at 37 °C. After 4 h, MTT supernatant was removed and DMSO (100 μL, Sigma, St. Louis, MO, USA) was added. The absorbance of formazan was measured at 490 nm (OD_490_). Cell viability was calculated from three independent experiments. The growth inhibitory rates of the complexes were calculated as (OD_control_ − OD_test_)/OD_control_ × 100%. IC_50_ values were calculated using the percentage of growth versus untreated control.

#### 3.2.3. Western Blot Analysis

HepG-2/ BGC-823/MCF-7/HCT-116 cells (1 x 10^6^ cells/well) were seeded on a 6-well plate with 2 mL DMEM/MEM/McCoy5A (10% FBS, 1% streptomycin, 5% CO_2_, and 95% air) at 37 °C for 24 h. After washing with PBS, the cells were incubated with compound **4a**/**4d**/**4e**/Cisplatin in 2 mL DMEM/MEM/McCoy5A medium (1% FBS, 1% streptomycin, 5% CO_2_, and 95% air) at 37 °C for 24 h. The cells were harvested by using RIPA lysis buffer with proteinase inhibitor cocktail to collect total protein lysate. The protein concentration in the total extract was quantitated by the BCA Protein Assay kit (562 nm). Western blot was carried out to analyze the expression of GSTs in the protein extract; protein (40 μg) extractions were separated by 12% SDS-PAGE (2 h) and transferred to PVDF membranes. The membrane was blocked with 5% defatted milk for 1 h and incubated 2 h at room temperature with the following primary antibodies: β-Actin (1:3000), CDK-4 (1:1000), BCL-2 (1:1000), MMP-9 (1:1000), and VEGFR-2 (1:500). After, the membrane was washed five times, followed by incubating with secondary antibodies goat anti-rabbit IgG (1:5000) and goat anti-mouse IgG (1:5000) for 1.5 h at room temperature. The relative density of each band was applied to a chemiluminescent indicator and was quantitatively detected.

#### 3.2.4. Molecular Docking

The 3D structures of ART, DHA, and compounds **4a**–**4e** were constructed using Chemoffice 2020 software (mol2. format) and downloaded from the PDB Crystal Protein Library (https://www.rcsb.org/, accessed on 19 November 2023) for the cell cycle protein CDK4 [40,41,42] (1gij), the apoptosis protein BCL-2 [43,44,45] (4man), the vascular endothelial growth factor VEGFR-2 [46,47,48] (4ase), and matrix metalloproteinase MMP-9 [49,50] (2ovz), four antitumor target proteins. The predicted binding patterns of the complexes formed between the target proteins and ART, DHA, and **4a** to **4e** were distinguished by a scoring function for the best set of conformations constructed during the sampling process. Based on the above theory, the four processed antitumor target proteins were sequentially docked to ART, DHM, and **4a** to **4e**, respectively, using LeDock.win32 software for semi-flexible molecular docking.

The closer the candidate conformation is to the conformation of the natural complex under ideal conditions, the lower the evaluation score derived from the scoring function, and the output of LeDock is the binding energy ΔG. Therefore, using the LeDock.win32 software molecular docking, the binding free energy of artemisinin, dihydroartemisinin, and compounds **4a**–**4e** was predicted to interact with the four target proteins, and combined with the scoring function of the original ligand of each target site as the threshold value. The target with the lowest binding energy to the target compound was selected. The Ki of the target compound for the potential target was further calculated according to the theoretical inhibition constant (Ki) formula.

Ki = exp[(ΔG × 1000)/(Rcal × T)]

(Note: ∆G in kcal·mol^−1^, T = 300 K, Rcal = 1.98719 cal·mol^−1^·K^−1^.

## 4. Conclusions

In summary, five artemisinin bivalent ligands were designed, synthesized, and tested for their antitumor activity against four human cancer cell lines, and verified by the “single ligand-multiple target” docking model. The experimental results showed that the antitumor activities of compounds **4a**, **4d**, and **4e** were better than artemisinin and dihydroartemisinin, and the IC_50_ of compound **4e** is 8.30 μmol/L against BGC-823 tumor cells. The evaluation of the linker in the preliminary SAR analysis is significant to the theoretical research and practical application of synthesizing artemisinin bivalent ligands.

## Figures and Tables

**Table 1:** Anti-proliferative activities of the target compounds against MCF-7, HepG-2, HCT-116, and BGC-823, four tumor cancer cell lines, in vitro.

Compd.	IC_50_ (μmol/L) ^a,b^			
	BGC-823	HepG-2	HCT-116	MCF-7
ART	34.82 ± 1.50	41.02 ± 1.64	31.47 ± 0.97	32.74 ± 0.45
DHA	29.64 ± 0.60	32.73 ± 0.99	41.69 ± 1.03	34.32 ± 0.65
**4a**	12.48 ± 0.60	11.75 ± 0.71	13.60 ± 0.74	18.24 ± 0.20
**4b**	>50	>50	>50	>50
**4c**	>50	>50	>50	>50
**4d**	16.31 ± 0.21	12.68 ± 0.87	21.68 ± 0.59	33.67 ± 0.47
**4e**	8.30 ± 0.14	40.31 ± 1.38	16.83 ± 0.30	24.27 ± 0.35

^a^ MTT methods, cells were incubated with corresponding compounds for 48 h; ^b^ IC_50_ values are shown as mean ± SD from the average of three replicates.

**Table 2:** Binding energies and amino acid residues involved in hydrogen bonding of bivalent ligands **4a**–**4e** within the binding sites of BCL-2, CDK-4, VEGFR-2, and MMP-9.

Compd.	Targets	Receptor Residues Involved in Hydrogen Bonding	Binding Energies (Kcal/mol)
**4a**	BCL-2	ARG143, TYR105	−5.64
	CDK-4	-	−6.37
	VEGFR-2	ASN923	−5.78
	MMP-9	HIS405, LEU187	−6.50
**4b**	BCL-2	ASN140, ALA97	−8.40
	CDK-4	-	−7.67
	VEGFR-2	-	−5.43
	MMP-9	ARG424, VAL398	−9.64
**4c**	BCL-2	ARG136, GLU133, GLY142	−7.31
	CDK-4	LYS123, THR89, HIS84	−6.46
	VEGFR-2	-	−3.94
	MMP-9	THR426, GLN402	−10.71
**4d**	BCL-2	-	−6.54
	CDK-4	-	−7.49
	VEGFR-2	ASP1046, GLU885, GLY922	−7.16
	MMP-9	GLN402, LEU418, TYR423	−8.70
**4e**	BCL-2	ARG143, GLY142	−6.49
	CDK-4	-	−6.94
	VEGFR-2	ASP1046	−6.83
	MMP-9	GLN402	−7.51

**Table 3:** Docking data of compounds and target proteins.

Compd.	CDK-4 (1GIJ)		BCL-2 (4MAN)	
	ΔG (kcal/mol)	K_i_ (μmol/L)	ΔG(kcal/mol)	K_i_ (μmol/L)
ART	−4.25	801.56	−3.64	2230.03
DHA	−4.65	430.07	−3.44	3118.96
**4a**	−6.37	22.88	−5.64	77.86
**4b**	−7.67	2.59	−8.40	0.76
**4c**	−6.46	19.68	−7.31	4.73
**4d**	−7.49	3.50	−6.54	17.21
**4e**	−6.94	8.80	−6.49	18.71
**Compd.**	**VEGFR-2 (4ASE)**		**MMP-9 (2OVZ)**	
	**Δ** **G (kcal/mol)**	**K_i_ (** **μmol/L)**	**Δ** **G (kcal/mol)**	**K_i_(** **μmol/L)**
ART	−4.13	980.29	−4.23	828.90
DHA	−4.27	775.12	−4.45	573.11
**4a**	−5.78	61.57	−6.50	18.40
**4b**	−5.43	110.74	−9.64	0.095
**4c**	−3.94	1348.24	−10.71	0.016
**4d**	−7.16	6.08	−8.70	0.459
**4e**	−6.83	10.58	−7.51	3.38

## Data Availability

Data are contained within the article and supplementary materials.

## Supplementary Materials

The following supporting information can be downloaded at: https://www.mdpi.com/article/10.3390/molecules29020409/s1, The synthesis of intermediates; The docking results of compounds with different proteins.
