# Supplementary material for: Design, Synthesis, and Antitumor Activity Evaluation of Artemisinin Bivalent Ligands
Source: Molecules. 2024 Jan 15;29(2):409. doi: 10.3390/molecules29020409 (PMC10818997; doi:10.3390/molecules29020409)
Supplement: Supplementary file 1 [file molecules-29-00409-s001.zip › molecules-2756241-supplementary.pdf]

## SUPPLEMENTARY MATERIAL

# Design, Synthesis, and Antitumor Activity Evaluation of Artemisinin Bivalent Ligands

**Hui Zhong [1§], Qi Jiang [1§], Cong Wu [1], Huanghe Yu [1,2], Bin Li [1,2], Xudong Zhou [1,2], Ronggeng Fu [1], Wei Wang [*1,2], Wenbing Sheng [*1,2]**

1 School of Pharmacy, Hunan University of Chinese Medicine, Changsha 410208, China

2 TCM and Ethnomedicine Innovation and Development International Laboratory, Hunan University of Chinese Medicine, Changsha 410208, China

§ These authors contributed equally to this work

* Correspondence: E-mail addresses: wangwei402@hotmail.com(W Wang), wbs626@126.com (W. Sheng).

## 1. Chemistry

The commonly used reagents were purchased from Sinopharm Group Chemical Reagent Co., Ltd and used without further purification. Thin-layer chromatography (TLC, silica gel HSGF254, Yantai Jiang You Silicone Development Co., Ltd. (Yantai, Shandong, China)) was used to monitor for completeness of the reaction visualized by UV light (λ = 254 nm or λ = 365 nm). The target compounds were purified by silica gel column chromatography. NMR spectra were recorded on the Bruker Avance III spectrometer at 600 MHz for $^1$H NMR and 150 MHz for $^{13}$C NMR, with tetramethylsilane (TMS) as the internal standard, and DMSO-d6, $CDCl_3$, and $CD_3OD$ were used as the solvent. Coupling constant (*J*) values were estimated in Hertz (Hz). Splitting patterns are designated as follows: s, singlet; br s, broad singlet; d, doublet; t, triplet; q, quartet; dd, doublet of doublet; m, multiplet. Mass spectra were measured on an LCMS 6400 Series Triple Quadrupole Mass Spectrometer (Agilent).

### 1.1 Synthesis of 11-N-artemisinin (1b)[1]

To a mixture of artemisinin **1a** (71 mg, 0.25 mmol) and ammonia (33%, 5 mL) in a mixture solvent of THF and $CH_3OH$ being stirred in cold hydrazine at -10 ~ -15°C. Reaction progress was monitored by TLC. After completion of the reaction, the mixture was removed by using a rotary evaporator to obtain a yellow solid. A round-bottomed flask was charged with the obtained product, $CH_2Cl_2$ and *p*-toluenesulfonic acid (0.3

mmol). The reaction mixture was stirred for 12 hours and was then washed with 5% $NaHCO_3$(aq.) and distilled water, respectively. The organic layer was separated, dried over anhydrous $Na_2SO_4$ and filtered. The solvent was removed under reduced pressure to afford a white solid, and was then recrystallized from ethyl acetate-hexane to obtain a white crystal **1b** 55.5 mg in 79% yield.

### 1.2 Synthesis of 11-N-methylpropionic acid methyl artemisinin (1c)[2]

In a 25 mL round-bottomed flask fitted with a reflux condenser were placed **1b** (28 mg, 0.1 mmol), methyl acrylate (26 mg, 0.3 mmol), NaOH (20 mg) and anhydrous THF (2 mL). The mixture was stirred magnetically at room temperature and monitored by TLC until **1b** disappeared completely. The reaction mixture was quenched by the addition of aqueous $NaHSO_3$ (5 mL, 0.1 mol/L), and was then extracted with $CH_2Cl_2$. The organic layers were combined and washed sequentially with saturated aqueous $NaHCO_3$, saturated salt water, then dried with anhydrous $Na_2SO_4$. The liquor obtained was distilled under reduced pressure to remove $CH_2Cl_2$ to obtain a colorless oily product, which was purified by silica gel chromatography to afford a white solid **1c** 29.4 mg in 80% yield.

### 1.3 Synthesis of 11-N-propionic acid-artemisinin (1d)[2]

To a mixture solution THF (5 mL) and $CH_3OH$ (3 mL) was added **1c** (38 mg, 0.1 mmol) and NaOH (10 mL, 1 mol/L). The reaction mixture was refluxed and monitored by TLC until the raw material point disappeared. After completion of the reaction, the pH was adjusted to 6-7 to give a solid. The obtained crude solid was filtered and dried to afford a white solid **1d** 32.8 mg in 93% yield.

### 1.4 Synthesis of 15, 20-phenylporphyrin-5, 10-phenylbenzoate (2b)[3]

A round-bottomed flask fitted with a reflux condenser were charged with methyl 4-formylbenzoate (2.95 g, 18 mmol), benzaldehyde (1.9 g, 18 mmol) and propionic acid (150 mL). The temperature of the flask was maintained at 140°C and stirred by dropwise addition of a mixture solution of freshly distilled pyrrole (36 mmol, 2.5 mL) and propionic acid (12.5 mL). After 1.5 h, the propionic acid was removed by distillation under reduced pressure and the residual solution was cooled to room temperature, and was then added ethanol (20 mL). The mixture was left standing

overnight and filtered to afford a black crude product. The obtained solid was chromatographed on silica gel ($CH_2Cl_2$) to afford 2.17 g of methyl 5,10-*p*-benzoate 15,20-phenylporphyrin (**2a**) as a purple solid in 33% yield.

A solution of compound **2a** (0.73 g, 1.00 mmol) in THF (50 mL) and NaOH (20 mL, 1 mol/L) was stirred at 66 °C and refluxed. Completion of the reaction was confirmed by TLC. After that, the pH was adjusted to 3 by progressively adding hydrochloric acid (1 mol/L) to obtain the crude solid. In order to obtain it completely pure, the crude substance was filtered, washed with distilled water and dried under vacuum at 80 °C for 12 h to afford a purple-brown solid **2b** 0.645 g in 92% yield.

**1.5 Synthesis of 10, 20-phenylporphyrin-5, 15-phenylbenzoate (3c)**[4]

A mixture of methyl 4-formylbenzoate (2.95 g, 18 mmol) and pyrrole (17.4 mL, 250 mmol) was degassed with $N_2$ for 2h. The solution was then added trifluoroacetic acid (1.4 mmol) and stirred at room temperature and protected from light for 4 h. The stirred reaction mixture was diluted with $CHCl_3$ (200 mL) and washed sequentially with aqueous NaOH (0.1 mol/L) and distilled water. The organic layers were dried over anhydrous $Na_2SO_4$ and evaporated under reduced pressure to remove the solvent and excess pyrrole to afford a viscous brown oil. The crude product was purified by column chromatography silica gel eluted with $CH_2Cl_2$ to give a light grey solid **3a** 2.07 g in 41% yield.

A mixture of **3a** (3.00 g, 10.7 mmol), benzaldehyde (1.13 g, 10.7 mmol) and $CH_2Cl_2$ (400 mL) was degassed with $N_2$ for 2 h. The solution was then added $BF_3 \cdot Et_2O$ (10.7 mmol) and stirred at room temperature and protected from light for 2 h. The stirred reaction mixture was added chloranil (4.5 g) to obtain the dull red solution and stirred overnight. After completion of the reaction, the mixture was added triethylamine (5 mL), concentrated and purified by flash column chromatography silica gel eluted with $CHCl_3$ to collect the purple-red band. The substituted porphyrin mixture was distillated in vacuo and chromatographed on silica gel (hexane and toluene) to give 1.25 g of methyl 5,15-*p*-benzo-10,20-phenylporphyrin (**3b**) as a purple solid in 16% yield. The structure of **3b** was identified by comparing the retention factor value with its reported in the literature.

A mixture of **3b** (1.00 mmol), THF (50 mL), and aqueous NaOH (20 mL ,0.1 mol/L) was stirred and refluxed at 60 °C. The pH was adjusted to 3 with hydrochloric acid (1 mol/L) while TLC analysis showed the complete consumption of the raw material. The mixture was filtered to give the crude product. The crude product was washed with distilled water and dried in vacuo at 80 °C for 12 h. The purple solid **3c** 0.639 g was obtained in 91% yield.

**1.6 Synthesis of artemisinin bivalent ligands (4a)**

In a 25 mL round-bottomed flask were placed **1d** (35 mg, 0.1 mmol), anhydrous DCM (5 mL), DCC (45 mg, 0.22 mmol), DMAP (27 mg, 0.22 mmol) and DHA (63 mg, 0.22 mmol). The mixture was protected by $N_2$ at room temperature and monitored by TLC. After completion of the reaction, the reaction mixture was quenched by the addition of distilled water (10 mL), extracted with ethyl acetate, washed sequentially with saturated saline and distilled water, then dried with anhydrous $Na_2SO_4$, distilled under reduced pressure to remove the solvent and purified by silica gel chromatography to afford **4a**.

**1.7 Synthesis of artemisinin bivalent ligands (4b)**

Following the same procedure for **4a**, **2b** (70 mg, 0.1 mmol), DCC (45 mg, 0.22 mmol), DMAP (27 mg, 0.22 mmol) and DHA (63 mg, 0.22 mmol) were reacted to give **4b**.

**1.8 Synthesis of artemisinin bivalent ligands (4c)**

Following the same procedure for **4a**, **3c** (70 mg, 0.1 mmol), DCC (45 mg, 0.22 mmol), DMAP (27 mg, 0.22 mmol) and DHA (63 mg, 0.22 mmol) were reacted to give **4c**.

**1.9 Synthesis of artemisinin bivalent ligands (4d)**

To a mixture pyridine-3,5-dicarboxylic acid (25 mg, 0.15 mmol) and anhydrous DMF (5 mL) was added EDCI·HCl (0.36 mmol), DMAP (0.36 mmol), DHA (0.36 mmol) and protected by $N_2$ at room temperature. The reaction was monitored by TLC. The slurry was quenched by the addition of distilled water (10 mL), extracted with ethyl acetate, washed sequentially with saturated saline and distilled water, dried with anhydrous $Na_2SO_4$, distilled in vacuo to remove the solvent and purified by silica gel

chromatography to obtain **4d**.

### 1.10 Synthesis of artemisinin bivalent ligands (4e)

Following the same procedure for **4d**, thiophene-2,5-dicarboxylic acid (25 mg, 0.15 mmol), EDCI·HCl (69 mg, 0.36 mmol), DMAP (44 mg, 0.36 mmol) and DHA (102 mg, 0.36 mmol) were reacted to give **4e**.

## Reference


1. Haynes, R.K.; Wong, H.-N.; Lee, K.-W.; Lung, C.-M.; Shek, L.Y.; Williams, I.D.; Croft, S.L.; Vivas, L.; Rattray, L.; Stewart, L.; et al. Preparation of N-Sulfonyl- and N-Carbonyl-11-Azaartemisinins with Greatly Enhanced Thermal Stabilities: in vitro Antimalarial Activities. *ChemMedChem* **2007**, *2*, 1464-1479, doi:https://doi.org/10.1002/cmdc.200700065.
2. Nguyen Le, T.; De Borggraeve, W.M.; Grellier, P.; Pham, V.C.; Dehaen, W.; Nguyen, V.H. Synthesis of 11-aza-artemisinin derivatives using the Ugi reaction and an evaluation of their antimalarial activity. *Tetrahedron Letters* **2014**, *55*, 4892-4894, doi:https://doi.org/10.1016/j.tetlet.2014.07.027.
3. Zhao, X.; Yuan, L.; Zhang, Z.-q.; Wang, Y.-s.; Yu, Q.; Li, J. Synthetic Methodology for the Fabrication of Porous Porphyrin Materials with Metal–Organic–Polymer Aerogels. *Inorganic Chemistry* **2016**, *55*, 5287-5296, doi:10.1021/acs.inorgchem.6b00274.
4. Miao, J.; Zhu, L. Hydrogen Bonding Induced Supramolecular Self-Assembly of Linear Doubly Discotic Triad Supermolecules. *Chemistry - An Asian Journal* **2010**, *5*, 1634-1641, doi:https://doi.org/10.1002/asia.201000017.

**2.Molecular Docking of compound and BCL-2, CDK-4, MMP-9 and VEGFR-2.**

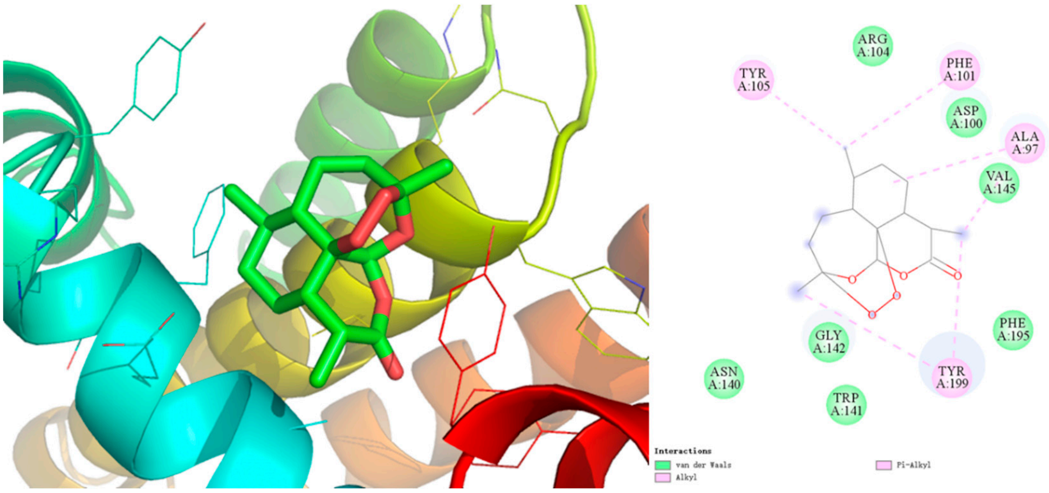


**Figure S1**. The docking results of **ART** with BCL-2 (PDB ID: 4MAN) is represented in 3D and 2D.

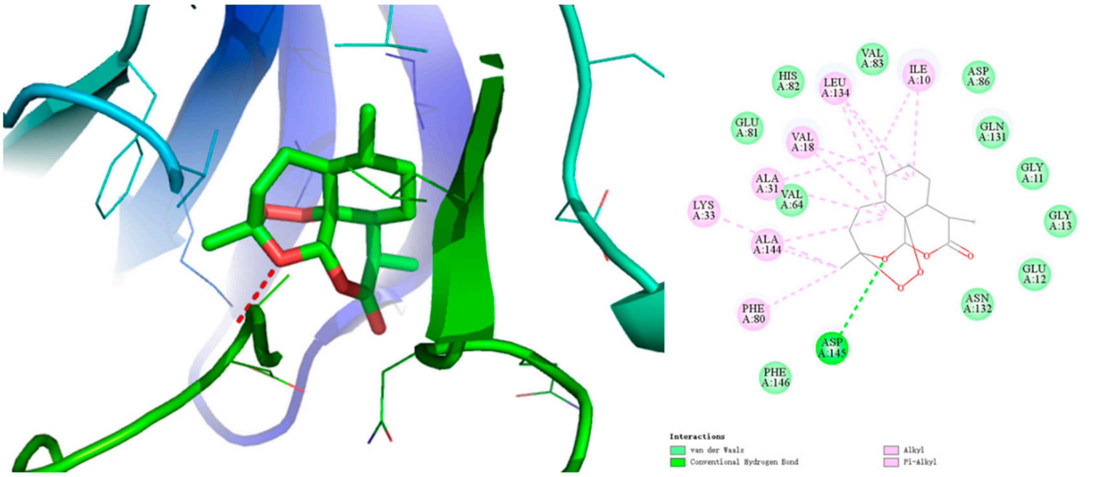


**Figure S2**. The docking results of **ART** with CDK-4 (PDB ID: 1GIJ) is represented in 3D and 2D.

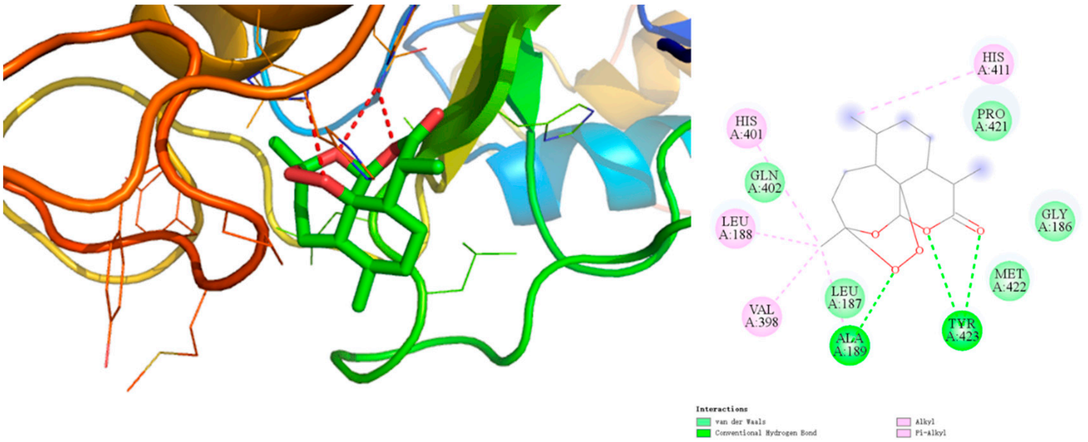


**Figure S3**. The docking results of **ART** with MMP-9 (PDB ID: 2OVZ) is represented in 3D and 2D.

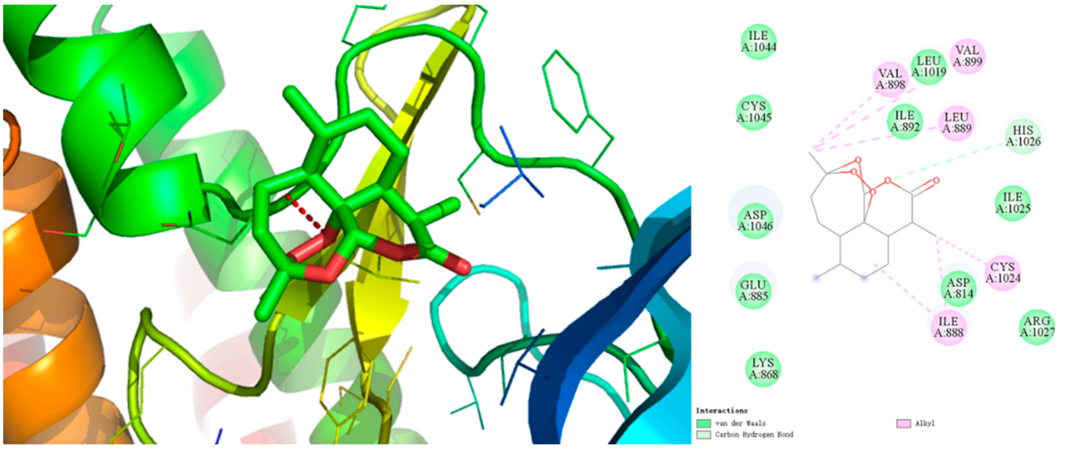


**Figure S4**. The docking results of **ART** with VEGFR-2 (PDB ID: 4ASE) is represented in 3D and 2D.

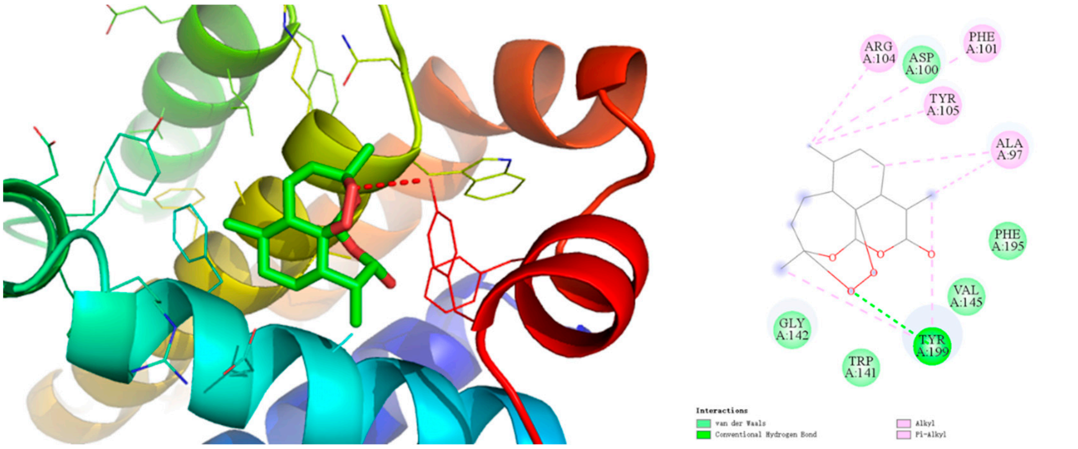


**Figure S5**. The docking results of **DHA** with BCL-2 (PDB ID: 4MAN) is represented in 3D and 2D.

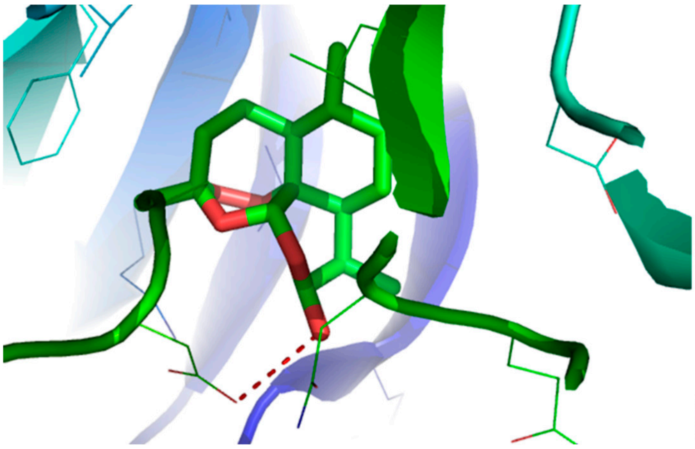


**Figure S6**. The docking results of **DHA** with CDK-4 (PDB ID: 1GIJ) is represented in 3D and 2D.

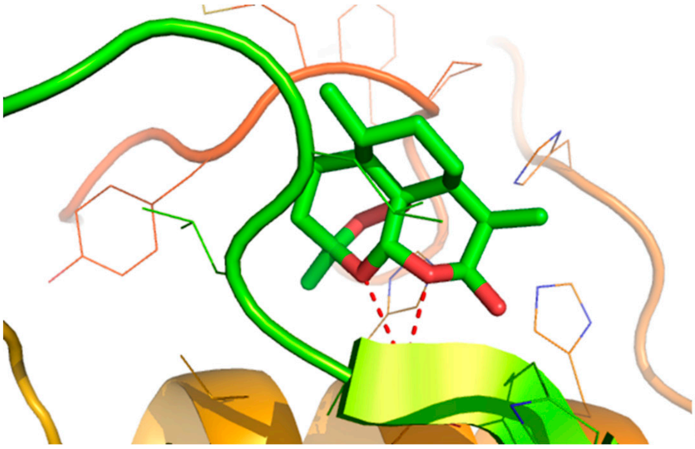


**Figure S7**. The docking results of **DHA** with MMP-9 (PDB ID: 2OVZ) is represented in 3D and 2D.

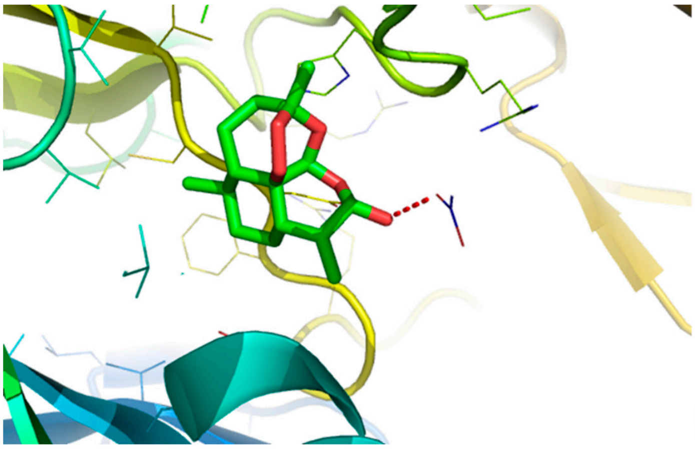


**Figure S8**. The docking results of **DHA** with VEGFR-2 (PDB ID: 4ASE) is represented in 3D and 2D.

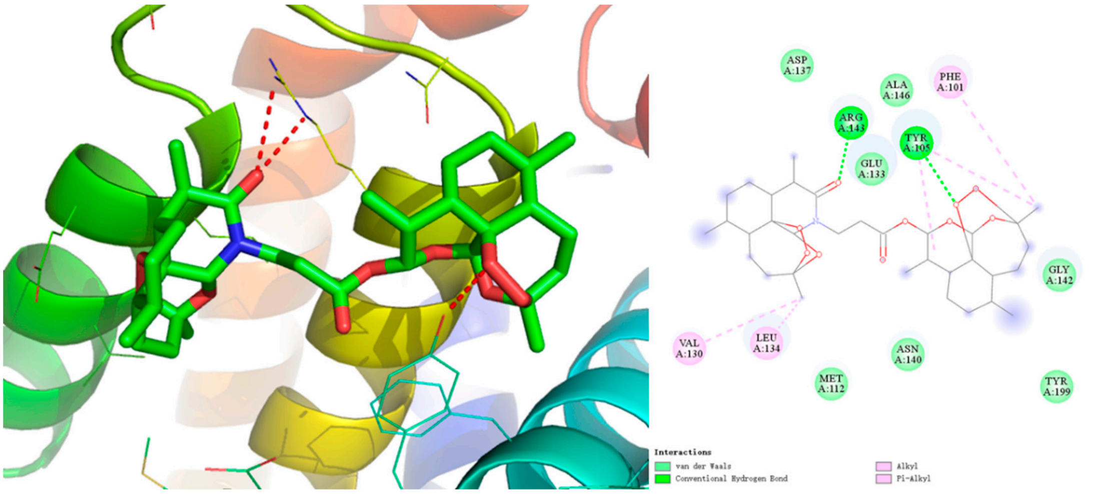


**Figure S9**. The docking results of **4a** with BCL-2 (PDB ID: 4MAN) is represented in 3D and 2D.

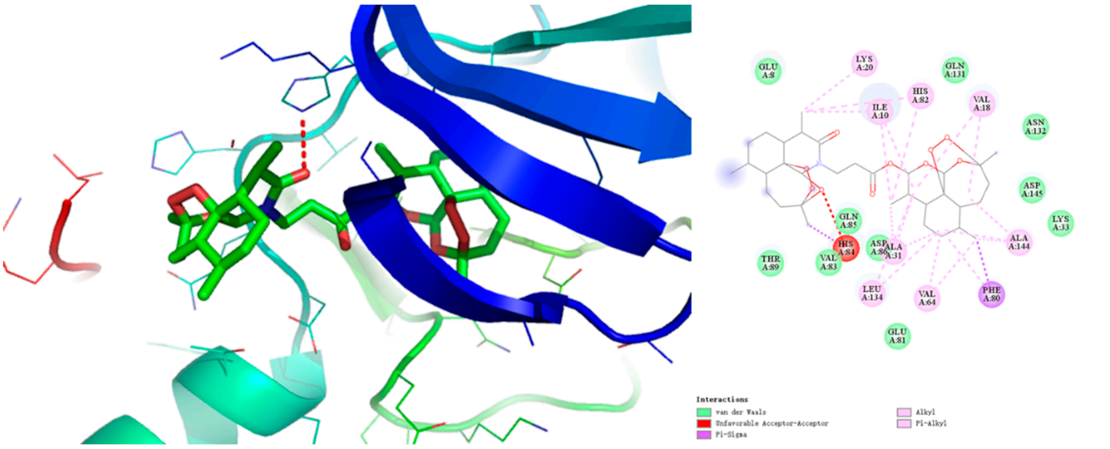


**Figure S10**. The docking results of **4a** with CDK-4 (PDB ID: 1GIJ) is represented in 3D and 2D.

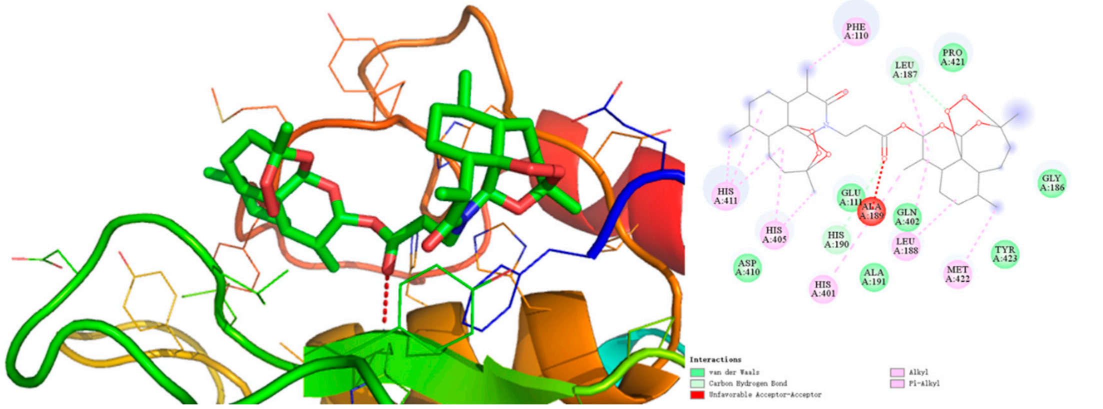


**Figure S11**. The docking results of **4a** with MMP-9 (PDB ID: 2OVZ) is represented in 3D and 2D.

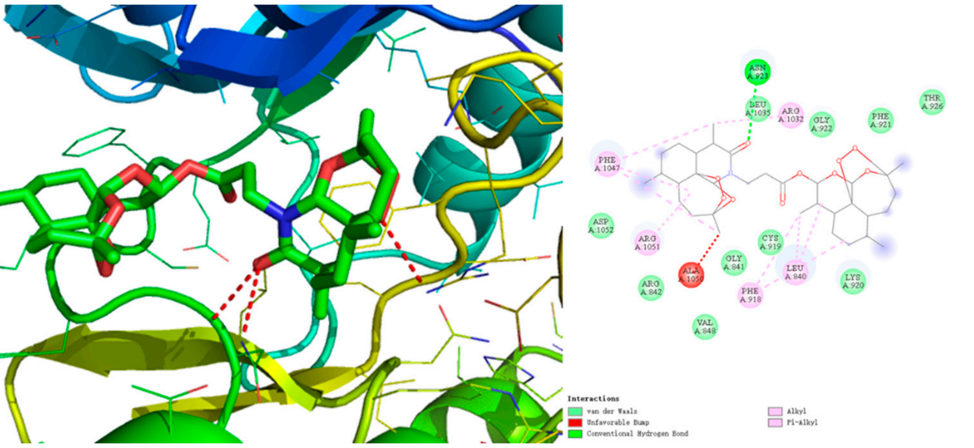


**Figure S12**. The docking results of **4a** with VEGFR-2 (PDB ID: 4ASE) is represented in 3D and 2D.

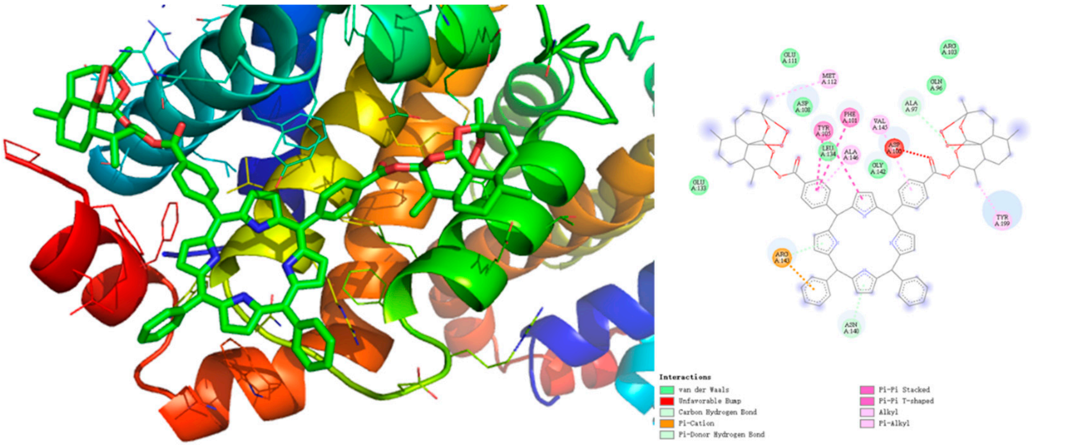


**Figure S13**. The docking results of **4b** with BCL-2 (PDB ID: 4MAN) is represented in 3D and 2D.

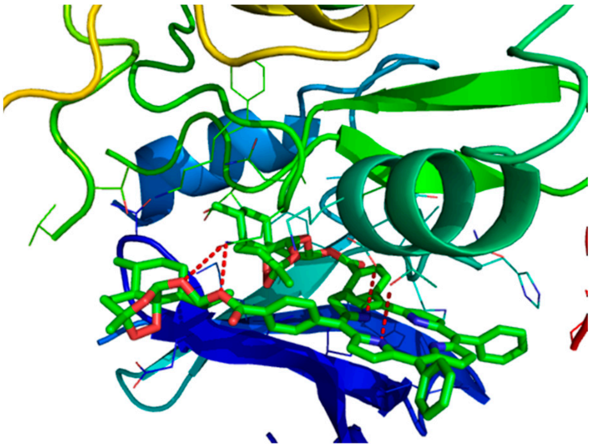

**Figure S14**. The docking results of **4b** with CDK-4 (PDB ID: 1GIJ) is represented in 3D.

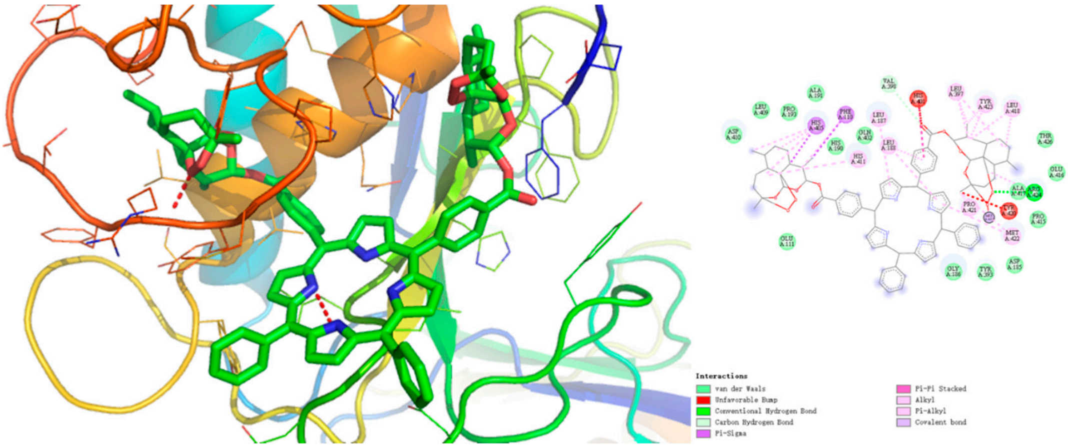


**Figure S15**. The docking results of **4b** with MMP-9 (PDB ID: 2OVZ) is represented in 3D and 2D.

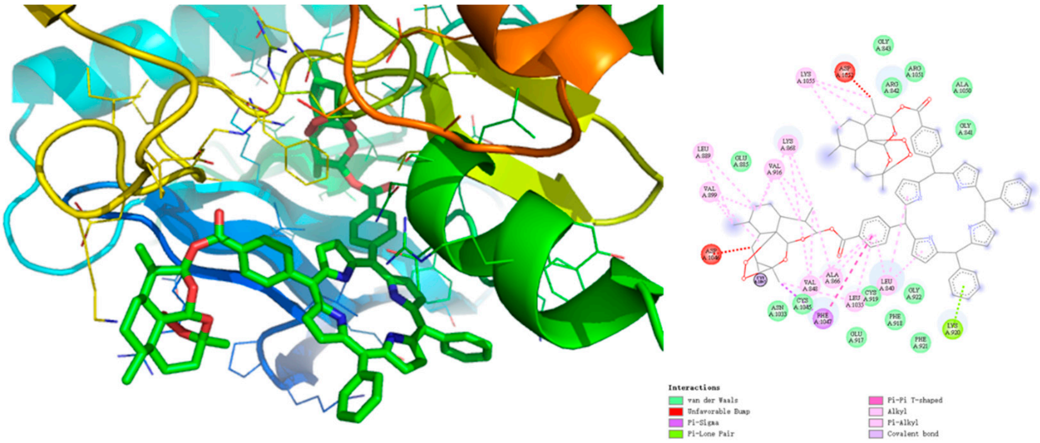


**Figure S16**. The docking results of **4b** with VEGFR-2 (PDB ID: 4ASE) is represented in 3D and 2D.

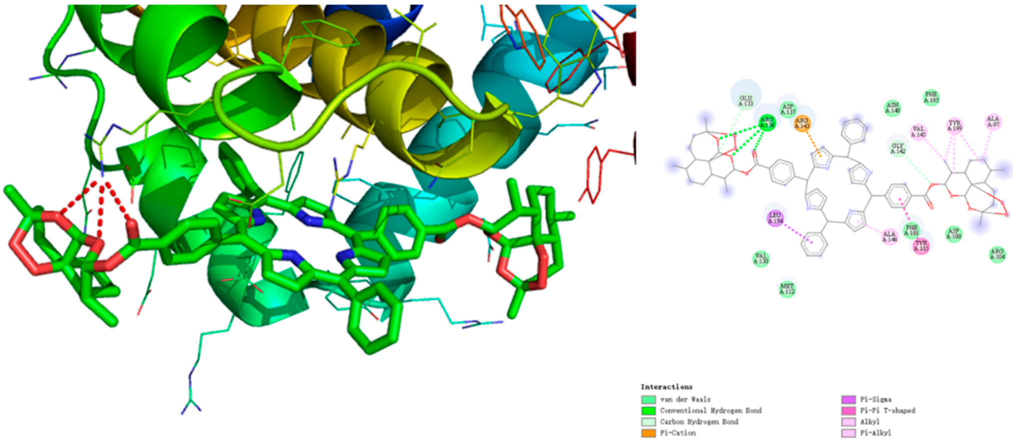


**Figure S17**. The docking results of **4c** with BCL-2 (PDB ID: 4MAN) is represented in 3D and 2D.

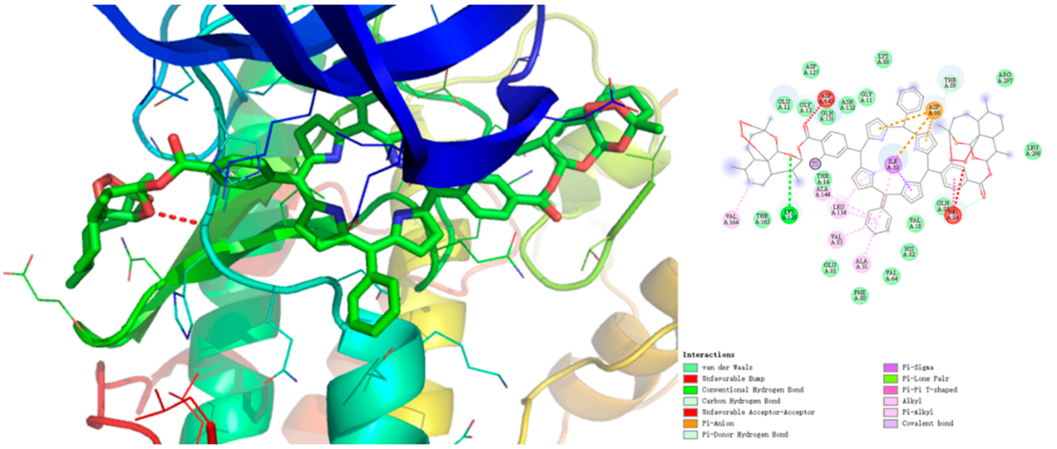


**Figure S18**. The docking results of **4c** with CDK-4 (PDB ID: 1GIJ) is represented in 3D and 2D.

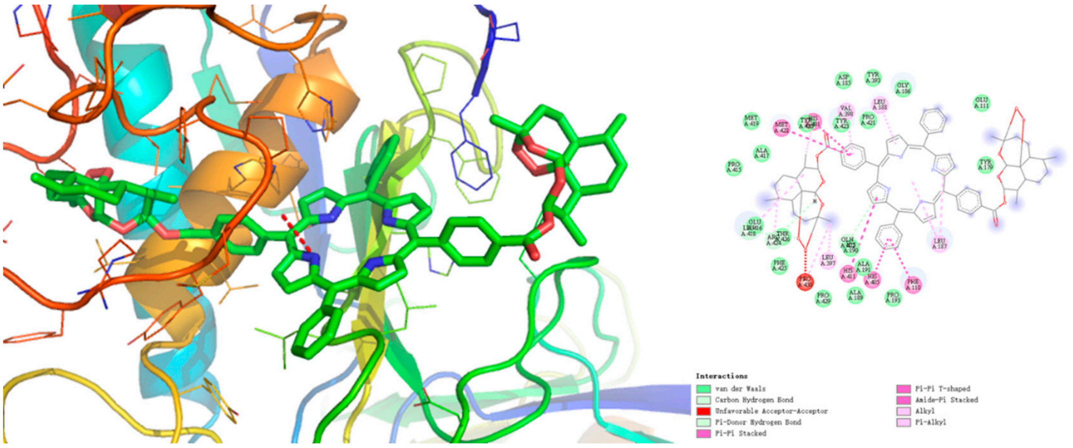


**Figure S19**. The docking results of **4c** with MMP-9 (PDB ID: 2OVZ) is represented in 3D and 2D.

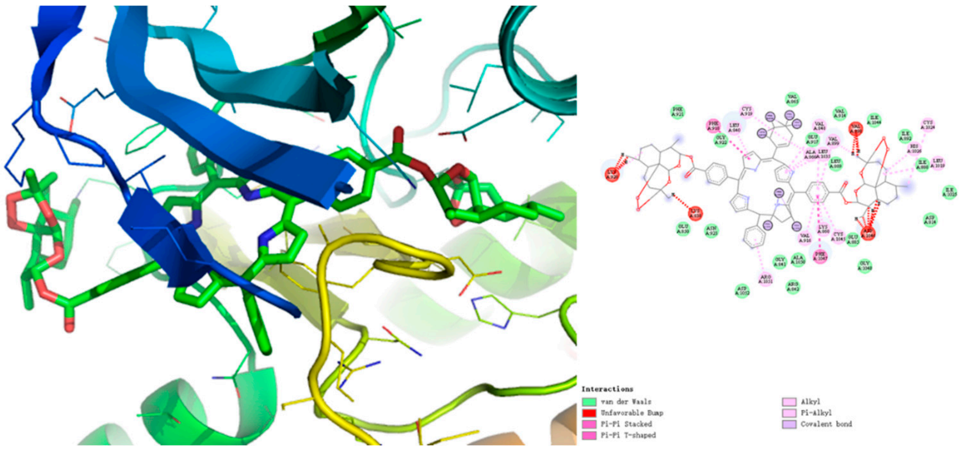


**Figure S20**. The docking results of **4c** with VEGFR-2 (PDB ID: 4ASE) is represented in 3D and 2D.

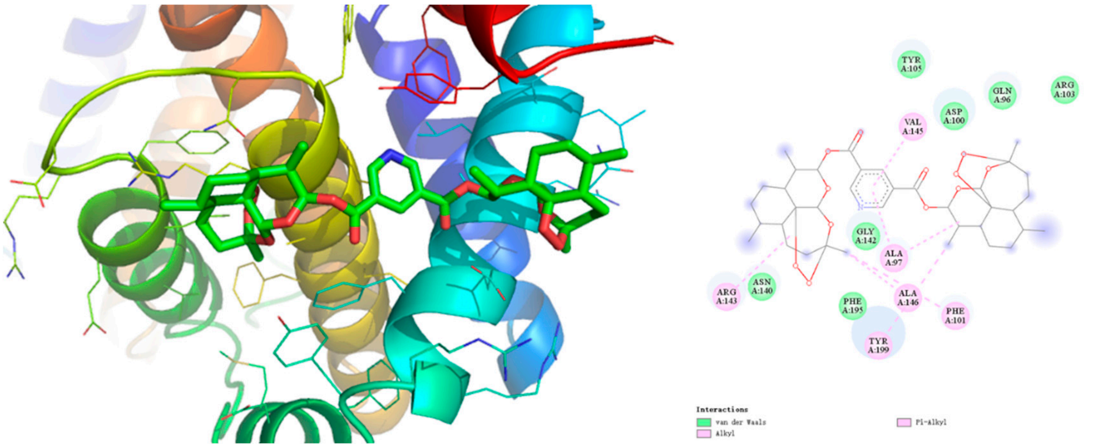


**Figure S21**. The docking results of **4d** with BCL-2 (PDB ID: 4MAN) is represented in 3D and 2D.

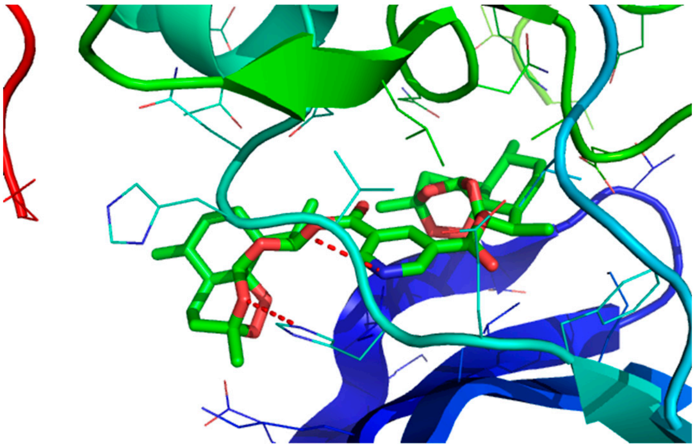

**Figure S22**. The docking results of **4d** with CDK-4 (PDB ID: 1GIJ) is represented in 3D.

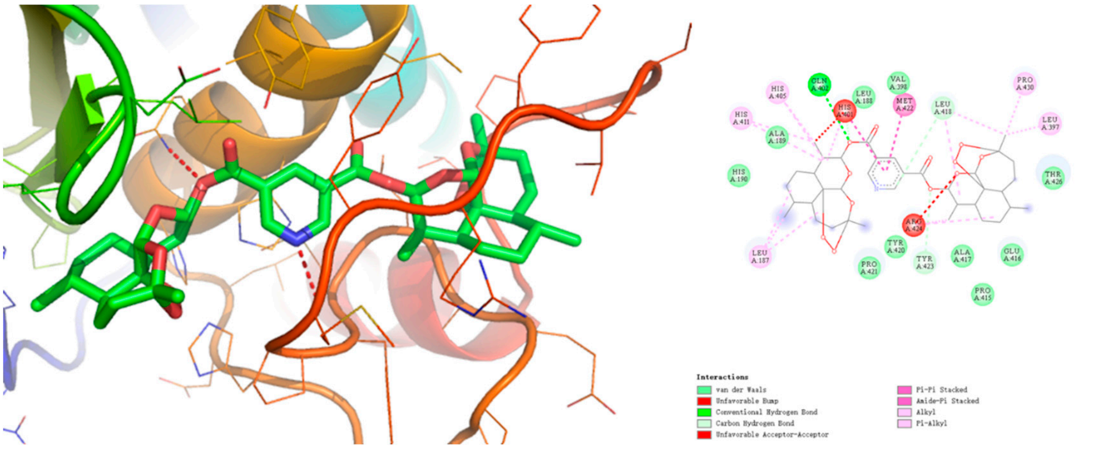


**Figure S23**. The docking results of **4d** with MMP-9 (PDB ID: 2OVZ) is represented in 3D and 2D.

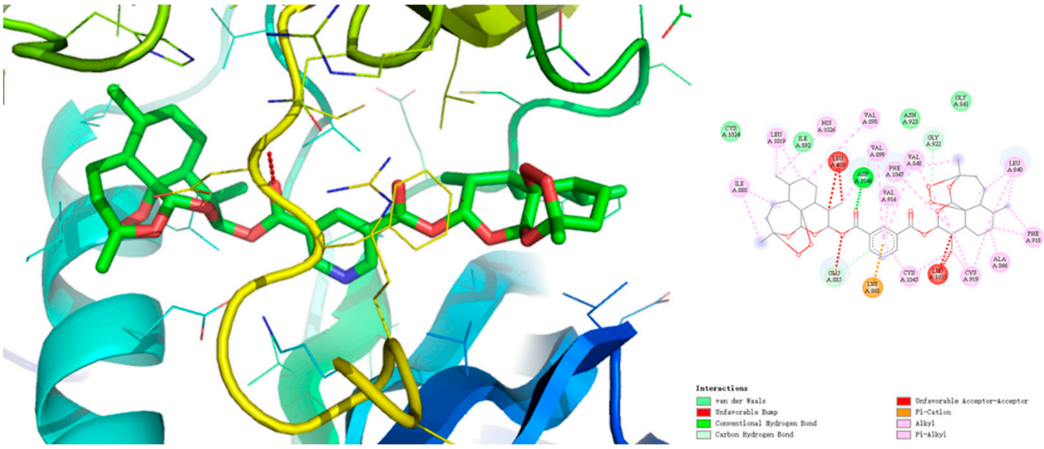


**Figure S24**. The docking results of **4d** with VEGFR-2 (PDB ID: 4ASE) is represented in 3D and 2D.

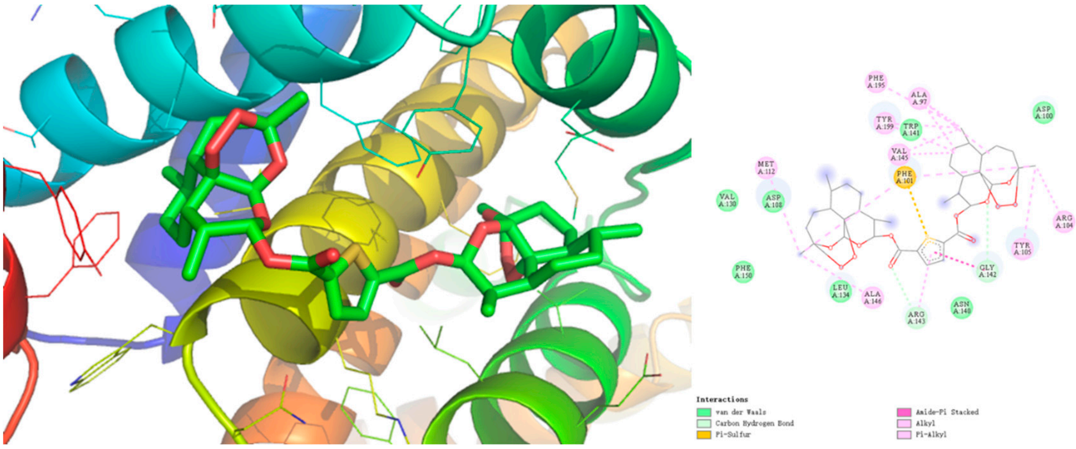


**Figure S25**. The docking results of **4e** with BCL-2 (PDB ID: 4MAN) is represented in 3D and 2D.

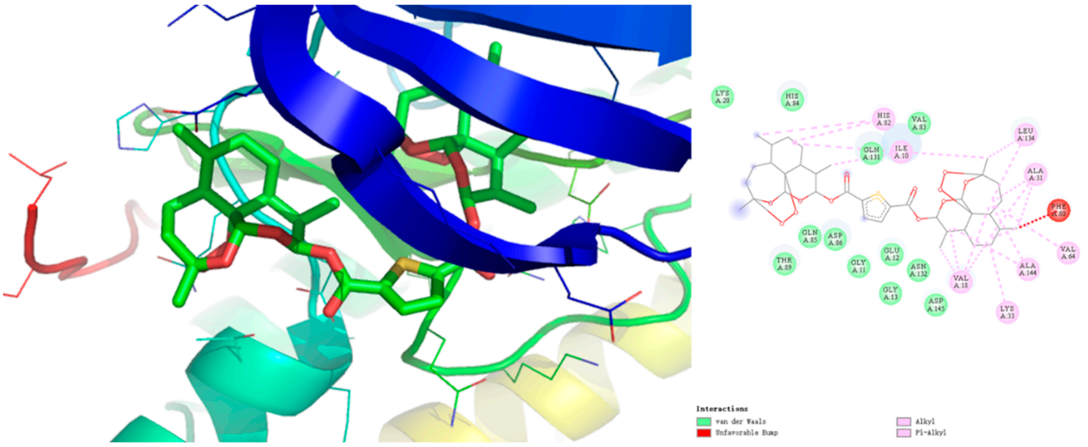


**Figure S26**. The docking results of **4e** with CDK-4 (PDB ID: 1GIJ) is represented in 3D and 2D.

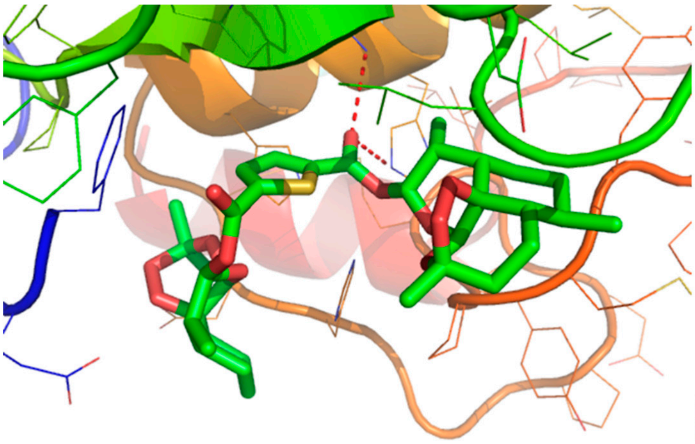


**Figure S27**. The docking results of **4e** with MMP-9 (PDB ID: 2OVZ) is represented in 3D and 2D.

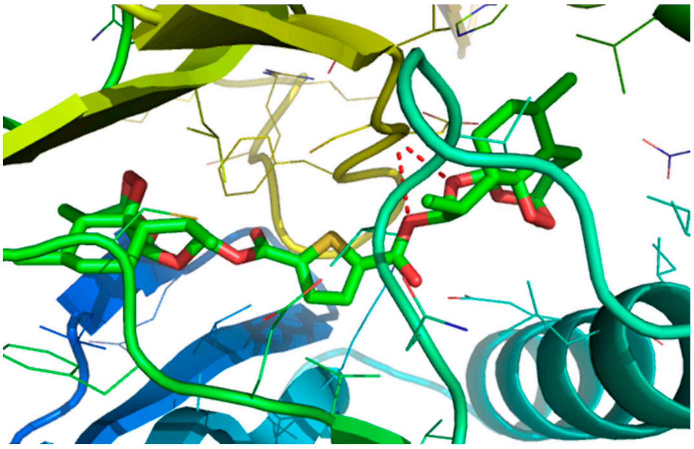


**Figure S28**. The docking results of **4e** with VEGFR-2 (PDB ID: 4ASE) is represented in 3D and 2D.

### 3. LC-MS spectrum of compounds 4a-4e.

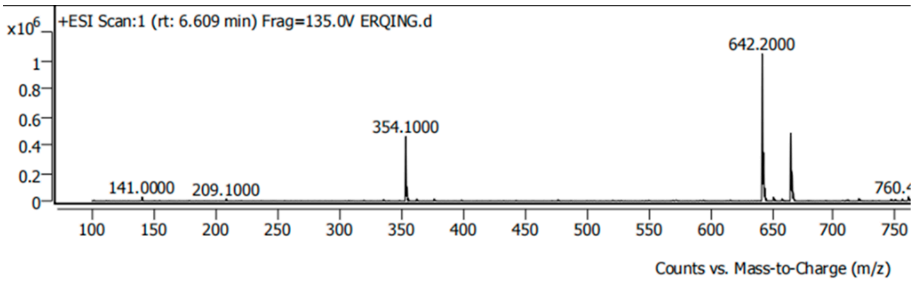


Spectrum Peaks

| m/z | Z | Abund | Abund % |
|---|---|---|---|
| 141.0000 | | 28405 | 2.72 |
| 209.1000 | | 14996 | 1.43 |
| 354.1000 | 1 | 458757 | 43.86 |
| 355.2000 | 1 | 95375 | 9.12 |
| 356.1000 | 1 | 18526 | 1.77 |
| 362.9000 | | 14073 | 1.35 |
| 377.1000 | | 14569 | 1.39 |
| 642.2000 | 1 | 1046037 | 100.00 |
| 643.3000 | 1 | 345078 | 32.99 |
| 644.2000 | 1 | 91329 | 8.73 |
| 645.3000 | 1 | 19010 | 1.82 |
| 651.2000 | 1 | 27300 | 2.61 |
| 652.3000 | 1 | 12632 | 1.21 |
| 658.2000 | | 15183 | 1.45 |

LC-MS spectrum of compound **4a**.

+ Scan:1 (rt: 6.571 min)

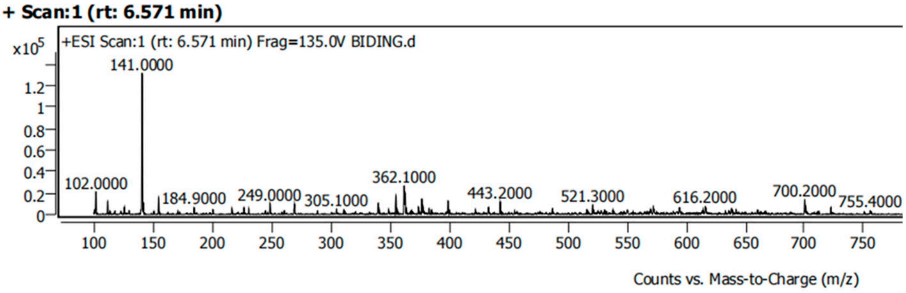


Spectrum Peaks

| m/z | Z | Abund | Abund % |
|---|---|---|---|
| 636.1000 | | 3663 | 2.78 |
| 638.3000 | | 5581 | 4.23 |
| 639.1000 | | 4378 | 3.32 |
| 642.2000 | | 4679 | 3.55 |
| 660.4000 | | 4392 | 3.33 |
| 662.4000 | | 2722 | 2.06 |
| 666.5000 | | 2858 | 2.17 |
| 667.4000 | | 2681 | 2.03 |
| 700.2000 | 1 | 13865 | 10.51 |
| 701.1000 | 1 | 6927 | 5.25 |
| 711.3000 | | 3089 | 2.34 |
| 722.3000 | | 6669 | 5.05 |
| 750.4000 | | 2820 | 2.14 |
| 755.4000 | | 3609 | 2.74 |
| 794.6000 | | 2838 | 2.15 |

LC-MS spectrum of compound **4d**.

- Scan:2 (rt: 4.550 min)

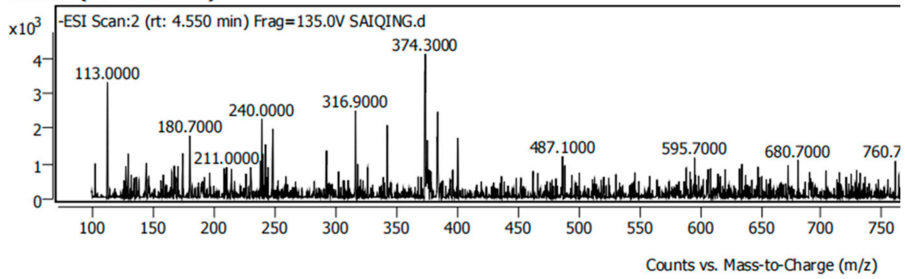


*Spectrum Peaks*

| m/z Z | Abund | Abund % |
|---|---|---|
| 690.2000 | 762 | 18.51 |
| 703.7000 | 809 | 19.65 |
| 714.7000 | 759 | 18.45 |
| 724.4000 | 711 | 17.29 |
| 728.9000 | 821 | 19.94 |
| 731.8000 | 738 | 17.93 |
| 760.7000 | 1075 | 26.14 |
| 763.6000 | 674 | 16.38 |
| 766.1000 | 680 | 16.52 |
| 767.5000 | 763 | 18.54 |
| 784.7000 | 697 | 16.94 |

LC-MS spectrum of compound **4e**.
